# Application of psychosocial models to Home-Based Testing and Counseling (HBTC) for increased uptake and household coverage in a large informal urban settlement in Kenya

**DOI:** 10.11604/pamj.2017.27.285.10104

**Published:** 2017-08-23

**Authors:** Patricia Oluoch, James Orwa, Fillet Lugalia, David Mutinda, Anthony Gichangi, Joseph Oundo, Mohamed Karama, Zipporah Nganga, Jennifer Galbraith

**Affiliations:** 1 Division of Global HIV/AIDS and Tuberculosis (DGHT), Centers for Disease Control and Prevention (CDC), Nairobi, Kenya; 2 Kenya Medical Research Institute (KEMRI), Nairobi, Kenya; 3 United States Army Medical Research Unit (USAMRU) Kericho, Kenya; 4 Jomo Kenyatta University of Agriculture and Technology, Nairobi, Kenya

**Keywords:** Home based testing and counseling, uptake, saturation, community participation and health belief model

## Abstract

**Introduction:**

Home Based Testing and Counselling (HBTC) aims at reaching individuals who have low HIV risk perception and experience barriers which prevent them from seeking HIV testing and counseling (HTC) services. Saturating the community with HTC is needed to achieve the ambitious 90-90-90 targets of knowledge of HIV status, ARV treatment and viral suppression. This paper describes the use of health belief model and community participation principles in HBTC to achieve increased household coverage and HTC uptake.

**Methods:**

This cross sectional survey was done between August 2009 and April 2011 in Kibera slums, Nairobi city. Using three community participation principles; defining and mobilizing the community, involving the community, overcoming barriers and respect to cultural differences and four constructs of the health belief model; risk perception, perceived severity, perceived benefits of changed behavior and perceived barriers; we offered HTC services to the participants. Descriptive statistics were used to describe socio-demographic characteristics, calculate uptake and HIV prevalence.

**Results:**

There were 72,577 individuals enumerated at the start of the program; 75,141 residents were found during service delivery. Of those, 71,925 (95.7%) consented to participate, out of which 71,720 (99.7%) took the HIV test. First time testers were (39%). The HIV prevalence was higher (6.4%) among repeat testers than first time testers (4.0%) with more women (7.4%) testing positive than men (3.6%) and an overall 5.5% slum prevalence.

**Conclusion:**

This methodology demonstrates that the use of community participation principles combined with a psychosocial model achieved high HTC uptake, coverage and diagnosed HIV in individuals who believed they are HIV free. This novel approach provides baseline for measuring HTC coverage in a community.

## Introduction

HIV testing and counseling (HTC) is the primary entry point to HIV prevention, care and treatment services [1]. Knowing one's HIV status provides those who are negative with the chance to remain HIV-free and those who are HIV positive, critical links to treatment, care, support and prevention interventions to reduce re-infection and transmission of HIV to others. There has been rapid expansion of HIV testing and counseling services in Kenya with changing models of service delivery moving from client initiated counseling and testing at static voluntary counseling and testing (VCT) sites to diagnostic testing and counseling (DTC) which was done at the discretion of the attending clinician for purposes of patient management to provider initiated testing and counseling (PITC) [2] where all clients visiting the health facility are offered services. When PITC was introduced in Kenya in 2008, the testing coverage was low with only 36% of Kenyans having ever tested for HIV [3]. Although an important approach for increasing coverage, PITC in health facilities reaches only clients who are sick and may miss those who perceive themselves to be HIV negative. Although the testing coverage has improved over the years, [4, 5], Kenya has not attained universal access to HIV testing. A major reason that hinders individuals from accessing HIV testing and counseling services in Kenya is low HIV risk perception [3]. Low HTC coverage has been associated with stigma [6, 7], lack of transport to testing sites and lack of time for testing and counseling [8, 9].

Home based testing and counseling (HBTC) is a community approach which takes HTC services to the individual with the aim of mitigating the challenges and barriers faced by clients. [10, 11]. Several studies demonstrated that HBTC was feasible and acceptable to clients from around the African continent and is also a cost effective strategy to increase access [12, 13]. A successful HBTC program achieves saturation of the target population with services [14]. Saturation is defined as obtaining at least 80% coverage of the target population [15]. Successful public health programs have embraced community participation and psychosocial models in helping communities change and adopt better strategies in dealing with health challenges [16]. In particular, community participation has been shown to be critical for successful HIV prevention behavior change interventions [17]. HBTC takes services to the individual in their home and community and should embrace the critical elements of successful community participation [16] which include: community entry, community mobilization and involvement, quality service provision and sensitivity and respect to the community's culture. Community entry is the first contact with community leaders and mapping process to promote understanding of community composition and dynamics. Community mobilization helps the community become motivated and involved to participate in identifying and solving their problems through dialogue [16]. This dialogue process promotes understanding of community dynamics for meaningful engagement, provision, uptake of quality service, referral and follow up to ensure linkage of individuals to additional appropriate services [18] including care and treatment for those who are diagnosed with HIV. Understanding of specific cultural influences on social norms and behaviors that foster healthy and safe lifestyles is key to community response in disease prevention for behavior change [19].

Behavior change communication is most successful when based on a social cognitive theory to increase health impact [20]. The Health Belief Model, (HBM), a cognitive psychosocial behavioral model with six constructs was designed to motivate individuals' health seeking behavior [21]. Four constructs are relevant in HIV prevention and include: 1) perceived susceptibility or risk; 2) perceived severity or seriousness or consequences; 3) perceived benefits or advantages if the behavior is changed and 4) lastly perceived barriers or costs of adopting alternative course of action. The degree to which these constructs are operationalized in counselling have the potential of increasing uptake of HTC services and influence behavior change. The degree to which the community embraces an intervention known as responsiveness; an inclusive approach of inquiry and action to foster effective program, is an important factor in increased utilization of services [22]. HBTC being a community service, its acceptance will be influenced by the community's views of about it and therefore use of sound community participation principles should obtain community saturation. Several studies on HBTC report its feasibility, acceptance and usefulness in identifying individuals who are HIV infected in Kenya [1, 23, 24] and in other African countries [11–13, 25, 26]. We did not find reports in literature on the aspect of community saturation with HTC services which HBTC should achieve. One of the envisaged benefits of HBTC is to increase access to HTC and achieve full coverage of the target population. This study incorporated the use of community participation principles and health belief model constructs in the HBTC program to increase uptake and achieve saturation with HTC services. This paper describes the processes we employed and outcomes for public health impact to achieve high uptake and measurable community coverage of HBTC services in Kibera, an informal settlement in Nairobi, Kenya.

## Methods

**Setting**: The Kibera program funded by President's Emergency Program for AIDS Relief (PEPFAR) through Centers for Disease Control and Prevention (CDC) was to increase HTC access by offering HBTC services to all eligible community residents with an aim of saturation of the entire slum dwelling. This public health intervention followed the successful implementation of a pilot project in two villages in Kibera that demonstrated the acceptability of HBTC [24] earlier. The program was implemented from August 2009 to April 2011 in Kibera, a low socio-economic neighborhood in the outskirts of Nairobi city with a reported population of 105,945 persons, low average income (US $ 20/month) and high rates of unemployment among young adults [27]. HIV associated risk behaviors include high alcohol consumption, prostitution and child labour and petty offences with HIV prevalence higher than the national average [24]. The households are tin and mud single rooms, very close to each other and arranged in distinct clusters [27] with each cluster forming a small administrative unit.

**Application of community participation principles and health belief model**: The model of community participation identified a three step process: a) community entry; b) community mobilization and involvement; and c) incorporation of cultural differences to promote community access and use of services.

### Community participation model

*Community entry and reaching the community*: a letter from the Ministry of Health (MOH) served as an introduction to the local administration facilitating smooth community entry and acceptance. Meetings were organized with community leaders at all levels to explain the aim of the project and used the constructs of health belief model to create demand for HIV services. The local administration facilitated and accompanied the project staff in a walk through the community for purposes of identifying landmarks, resources, boundaries and for general introduction to the residents. We carried out mapping of households involving assigning them numbers in a chronological order and all members of each household enumerated. A master list of all the residents was generated to determine the target population to calculate coverage and also facilitate follow-up among those missed at initial visit.

*Community mobilization and involvement*: community members were recruited for short term contracts for mobilization, household marking and census; others were engaged as security marshals to mitigate insecurity. The recruitment was guided by setting the eligibility criteria including: a minimum education standard, good conduct and ability to communicate with residents. The village elders vetted the applicants for suitability. The village mobilizers were given a three day training covering basic facts on HIV and the need and benefits of knowing ones HIV status. Case presentations and role plays on how to approach fellow residents were the dominant training techniques along with presentation of basic facts about HIV. Those that displayed proficiency in knowledge and demonstrated the ability to share the information with others were enrolled. Community mobilization included organized meetings, known as chief's "barazas", to discuss community concerns. Roadshows; forums which combine music, puppetry, dance from caravans mounted with public address system traversed through the villages with messages about HIV risks, the HBTC service and the need to know ones HIV status were used. The mobilizers further visited residents individually on a one-on-one encounter and shared the benefits of taking the HIV test. The local radio station was used to air messages about need to know HIV status and brochures containing HIV prevention messages were distributed to the residents.

**Consideration of cultural differences**: HTC service providers were trained on household dynamics and respect for the culture of the clients and how to identify specific risk behaviours. Specific attention was given to dressing and language to gain community acceptance. The language used varied with both age and cultural groups and service providers adapted appropriately. This was specifically important when discussing issues around sexual activities, for example younger participants used the expression of "getting into the box" to mean that one consented to casual sex. Service providers had to adopt flexible working schedules which included weekends and early mornings to capture clients who were away during first visit. The service providers regularly held group debriefs with their supervisors to share challenging situations encountered and brainstorm on solutions.

**HTC service provision and application of health belief model**: NASCOP trained and certified HTC service providers with interest to work in informal settlements were recruited and retrained on the program approaches. Topics included community mobilization, home entry process, family dynamics, use of the constructs of HBM and respect for the client's culture as well as quality control procedures to ensure correct results. Other topics addressed included screening for alcohol and drug abuse, screening for tuberculosis infection to facilitate identification of unmet needs and offer proper referral. The staff were also retrained on interviewing techniques, data recording and keeping. The service providers visited individual households and used four constructs of Health Belief Model (HBM) to examine the individuals' current HIV knowledge, beliefs and behaviors around HIV to assess perceived HIV vulnerability. Perceived HIV risk was assessed by asking leading questions based on individual circumstances and behaviour identified to help the individual increase their perception of HIV vulnerability. The second HBM construct of perceived severity was used to explore whether the individual and community recognized and understood the consequences of HIV infection. We provided education in simple terms and language, dispelled myths and rumors to increase perception of severity. The construct of perceived benefits of alternative action was used in discussions about advantages of knowledge of individual HIV status including available interventions that reduce the impact of HIV eg availability of free medications that if taken properly, improve the health of the individual and prevent transmission. Lastly, we explored the construct of perceived barriers, including costs and disadvantages of adopting new behavior or alternative course of action e.g condom use, reduction in number of sexual partners and linkage to care and treatment services. Individuals were given facts and education around HIV preventive behavior and their benefits. HIV testing and counseling was offered to all eligible and consenting individuals, following the National HTC guidelines [28] and observing the principles of HTC of consenting, counseling, confidentiality and respect for human rights. Serial testing was done using DetermineTM (Abbott Laboratories, USA) as the first test (this takes 15 minutes to be ready for reading) and confirmation for those reacting positive was done using BiolineTM. Discordant results on the two kits were confirmed using Uni-gold (Trinity Biotech PLC, Ireland) as a tie breaker; this takes 10 minutes. There was provision for sending inconclusive results following this algorithm to the national reference laboratory for confirmation (data not included in this analysis).

**Data management and analysis**: Data was collected alongside service provision from each participant using a structured questionnaire (adopted from the national HTC tool). Each participant was assigned a unique code for privacy protection. Data was collected on demographic characteristics, reasons for testing and not testing and type of HTC service provision, (as individuals, couples or families) and HIV test results were recorded. Completed forms were checked by the supervisor on a daily basis for completeness and accuracy and delivered to the data manager based off site. The forms were reviewed for any omission or errors and then scanned using verity Teleform software into a password protected access database. Incomplete forms were returned to the respective service provider for further verification. All consenting community members were included in the analysis, no sampling was conducted. Descriptive statistics were applied to describe the socio-demographic characteristics of the participants. Categorical variables were described with frequency tables including cell counts and corresponding percentages. HIV prevalence was determined by simple percentage calculation of those testing HIV positive out of the total testing for HIV overall, by gender and by either first time or repeat testing.

**Ethical considerations**: This project was approved by the Kenya Ministry of Health, the office of Associate Director for Science at the US Centers for Disease Control and Prevention, Atlanta and Kenya Medical Research Institute Ethical review board. Informed consent was obtained from each eligible participant above the age of 18 years as per the national HTC guidelines. Mature minors, below the age of 18 years, defined as pregnant, had a child, living in a consensual sexual partnership or married, provided consent per the national guidelines. Parents or guardians provided consent for testing of children below 18 years of age; children old enough to understand the procedure provided assent as per the national guidelines [28].

## Results

**Community participation and involvement**: A total of 794 residents were employed as, short term hires working as community mobilizers, household markers, census crew members and security marshals. Community members worked collaboratively with HTC service providers and contributed to the high service uptake. The roadshows pulled large crowds and following.

**Characteristics of the participants**: A total of 71,925 residents (males (49.5%) and females (50.5%)) participated in the project. The majority (61.6%) were aged between 19-34 years. There were slightly more married individuals 33,461 (46.62%) than the single 31,363 (43.7%) with a few reporting widowhood 1,499 (2.1%). Most of the residents reported having attained primary level of education 38,453 (53.7%) followed by secondary level of education (35.5%) and only 3.4% had no formal education. The majority (31.8%) of the residents reported being engaged in skilled jobs followed by unskilled jobs (28.1%) and a minority (3.4%) having professional jobs, while 14.7% were students. A good proportion (22%) reported no occupation. The majority (79.4%) of these residents reported non condom use (Table 1).

**Table 1:** Demographic characteristics of participants by HBTC uptake in Kibera slums

		Testing status		Unadjusted OR	Adjusted OR
	Total	Tested for HIV	Did not test for HIV		
variable	N (%)	n (%)	n (%)	OR (95% CI)	OR (95% CI)
**Sex**					
Male	35614 (49.50)	35501(99.68)	113 (0.32)	ref	
Female	36328 (50.50)	36219(99.70)	109 (0.30)	1.058 (0.813-1.377)	0.857 (0.586-1.254)
**Have you ever had an HIV test done**					
Repeat testers	43821 (60.91)	43746 (99.83)	75 (0.17)	3.065 (2.320-4.050)	1.586 (1.094-2.298)
New testers	28121 (39.09)	27974 (99.48)	147 (0.52)	ref	
**Categories of Age in years**					
0-12	935 (1.30)	932 (99.68)	3 (0.32)	ref	
13-18	10768 (15.00)	10747 (99.80)	21 (0.20)	1.647 (0.490-5.533)	0.896 (0.193-4.163)
19-24	23060 (32.13)	23007(99.77)	53 (0.23)	1.397 (0.436-4.480)	0.711 (0.145-3.476)
25-34	21382 (29.79)	21331 (99.76)	51 (0.24)	1.346 (0.419-4.322)	0.855 (0.167-4.378)
35-44	9220 (12.84)	9205 (99.84)	15 (0.16)	1.975 (0.571-6.835)	1.502 (0.269-8.406)
45-54	4448 (6.20)	4429 (99.57)	19 (0.43)	0.750 (0.222-2.541)	0.562 (0.104-3.047)
55-64	1383 (1.93)	1378 (99.64)	5 (0.36)	0.887 (0.211-3.721)	0.916 (0.128-6.543)
65+	584 (0.81)	530 (90.75)	54 (9.25)	0.032 (0.010-0.102)	1.012 (0.081-12.886)
**Occupation**					
None	15787 (22.00)	15757 (99.81)	30 (0.19)	ref	
Unskilled	22822 (31.81)	22767 (99.76)	55 (0.24)	0.668 (0.293-1.522)	0.749 (0.275-2.041)
Skilled	20098 (28.01)	20053 (99.78)	45 (0.22)	0.848 (0.534-1.347)	0.828 (0.485-1.412)
Professional	2464 (3.43)	2457 (99.72)	7 (0.28)	0.874 (0.507-1.505)	1.050 (0.477-2.314)
Student	10580 (14.75)	10557 (99.78)	23 (0.22)	0.788 (0.505-1.230)	0.842 (0.513-1.381)
**Education**					
No formal	1937 (2.71)	1931 (99.69)	6 (0.31)	ref	
Primary	38453 (53.71)	38362 (99.76)	91 (0.24)	1.310 (0.573-2.996)	1.320 (0.501-3.480)
Secondary	25419 (35.50)	25375 (99.83)	44 (0.17)	1.792 (0.763-4.210)	1.782 (0.642-4.950)
Tertiary	5785 (8.08)	5768 (99.71)	17 (0.29)	1.054 (0.415-2.678)	0.993 (0.306-3.224)
**Marital status**					
Single	31363 (43.70)	31304 (99.81)	59 (0.19)	ref	
Married monogamous	33461 (46.62)	33373 (99.74)	88 (0.26)	0.715 (0.514-0.994)	0.585 (0.328-1.043)
Married polygamous	2216 (3.09)	2209 (99.68)	7 (0.32)	0.595 (0.271-1.304)	0.406 (0.157-1.050)
Widowed	1499 (2.09)	1497 (99.87)	2 (0.13)	1.411 (0.344-5.5779)	1.124 (0.371-3.403)
Separate/Divorced	3230 (4.50)	3226 (99.88)	4 (0.12)	1.520 (0.552-4.187)	1.131 (0.244-5.244)
**Condom use in the last 3 months with a steady partner**					
Never	35477 (79.4)	35217 (99.27)	260 (0.73)	0.299 (0.073-1.218)	0.223 (0.030-1.637)
Sometimes	4067 (9.14)	4040 (99.34)	27 (0.66)	0.262 (0.057-1.196)	0.178 (0.022-1.419)
Always	3105 (6.98)	3093 (99.61)	12 (0.39)	ref	
No sex last 3 months	214 (0.48)	212 (99.07)	2 (0.93)	0.356 (0.086-1.464)	0.155 (0.013-1.916)
No steady partner	1628 (3.66)	1624 (99.75)	4 (0.25)	0.313 (0.057-1.713)	0.188 (0.019-1.834)

**Figure 1:**
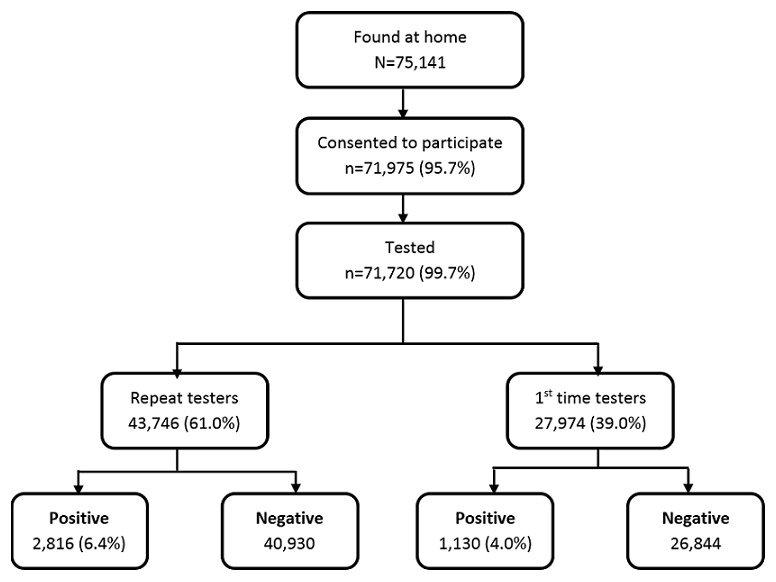
Coverage and uptake of HBTC in Kibera slums, Nairobi\

**HIV testing uptake and slum coverage**: The informal settlement census and household mapping identified a total of 72,577 eligible participants (Figure 1). However during service delivery, 75,141 residents were found at home and of those, 71,925 ((95.7 %) (Males 52%)) agreed to participate in the counselling and education session. Out of the 71,925 who agreed to participate, a total of 71,720 (99.7%) agreed to test for HIV. Of those who tested, 27,974 (39%) were first time testers and 43,746 (60.99%) had ever tested for HIV.

**HIV prevalence**: A total of 3,949 (5.5%) participants tested positive for HIV. There was significantly higher HIV prevalence rates (6.4%) among those ever tested for HIV than those testing for the first time (4.0%) (p < 0.0001). Women were more likely to test positive for HIV (7.4%) than men (3.6%) (Figure 1).

**Reasons for HIV testing**: Participants were asked reasons why they accepted or declined testing for HIV. The majority (61.1%) of the participants accepting testing reported they "wanted to know their HIV status and plan for future" followed by "because the service provider had gone to the household and recommended the test (24.6%)". Reasons for not testing were varied including; "already know their status (16.4%), "too old to get HIV" (9.3%) and "too busy" (8.2%) (Table 2).

**Table 2:** Reasons for acceptance or declining to take the HIV test by residents of Kibera slums, Nairobi

Reasons for declining the HIV test	n	%
Already know status	30	16.4
Too old to get HIV	17	9.3
Think test result may be incorrect(does not trust test kits)	15	8.2
Against religious beliefs	13	7.1
Too busy	9	4.9
Spouses does not want him/her to be tested	8	4.4
Fear of counseling procedures	8	4.4
Prefer to test away from home	7	3.8
Spouses does not want him/her to be tested	8	4.4
Does not think she/he has HIV or is at risk of getting HIV	6	3.3
Afraid to know results	6	3.3
Wants spouses/partner for test	6	3.3
Parent did not consent	6	3.3
Prefer to test without partner present in home	6	3.3
Does not think she/he has HIV or is at risk of getting HIV	6	3.3
Fear of testing procedures: finger prick, rapid test	3	1.6
Other reasons	30	16.4
**Reasons for accepting the HIV test**	**n**	**%**
Plan for future/ to know HIV status	42484	61.1
Because the provider recommends	17113	24.6
Wants to confirm previous test result	4380	6.3
Feel more knowledgeable about HIV now than before	1753	2.5
Partner risk behaviour	575	0.8
More privacy at home than at a VCT	469	0.7
Feel unwell	382	0.5
Plan to get pregnant	314	0.5
Don't want to or cant travel to a VCT	194	0.3
Mother is HIV+/deceased, for children	112	0.2
Plan to get married	80	0.1
Re-union with spouse/sex partner	57	0.1
Other reasons	1572	2.3

## Discussion

The Kenyan National Community Based Testing and Counseling Services operational manual recommends that a successful HBTC program should achieve population saturation of the targeted geographical area. Saturation in this context is defined as reaching and providing HTC services to at least 80% of the population in the targeted area [14]. The population coverage with HBTC services in the Kibera program surpassed the targeted population at the census by 3.4% as there were an additional 2,564 persons identified during implementation of services. Overall uptake (where uptake is the number of individuals who actually took the HIV test) of testing was at 99.7%. This demonstrates a higher acceptability of HBTC than (previously published reports ranging from 65.9% [29] through to 69% [30, 31] and the highest ever reported coverage being 81.7% [24] in a controlled pilot research program in the same area. We attribute this remarkable uptake of services to community participation and use of psychosocial model to guide this implementation. The important step of mapping and obtaining the census of the population provided the denominator which allowed for monitoring of progress and calculation of the coverage at the completion of the implementation. Our findings on this community's response are contrary to findings of fear of stigma reported in literature as a hindrance to access [6, 7]. Community participation potentially reduced stigma around HIV testing. A fair distribution of benefits among community members is a substantial incentive for community participation [32].

The program recruited 794 members of the community for short term paid employment in a transparent manner by involving the community members in the recruitment process. Use of a wide range of methods of community mobilization beyond just hanging fliers and posting public notices [33] including roadshow edutainment, community meetings and one to one contacts with community members created demand for services and may have promoted uptake too. The use of community members who are known to the community, as village mobilizers enhanced access to individuals and played an important part in reaching residents who were not at home at the initial visit. The mobilizers identified and made appointments for the re-visits by service providers at the client's convenience thus increasing coverage and community response. This program was sensitive to the cultural diversity in Kibera slums and each village was approached uniquely e.g through use of appropriate dress, language and even time of service provision by the service providers and sensitivity to diverse cultural practices. Culture not only refers to race and/or ethnicity, but also to unique characteristics of a community's population related to factors such as geography, age, gender, language, local history and economics [19] which the program was sensitive to. Our findings and experience with community participation in this program are consistent with earlier reported results in other primary health care programs for disease control where community participation was the central strategy including; control of dengue fever in urban Thailand [34]; schistosomiasis control in Kenya [35]; control of Chagas disease in Brazil [36] and HIV/AIDS control in an African community in Toronto [37]. The use of health belief model helped individuals explore their HIV risks, vulnerabilities, barriers and advantages of changed behavior at individual level and may have fostered in-depth understanding and created individual demand for wanting to know their HIV status yielding a testing coverage of 99.7% of those who agreed to participate in the sessions.

Targeting HIV education based on the four constructs of HBM; perceived vulnerability which is the individuals perceived susceptibility to a disease; perceived severity, referring to the seriousness of the disease and its consequences as perceived by the individual, perceived benefits if behavior is changed and barriers or costs of adapting an alternative course of action to mitigated psychosocial barriers to uptake of testing. We used the constructs of beliefs and attitudes as proximal determinants of behavior as the guiding principle and applied to community and individual circumstances to explore their perception of vulnerability to HIV, explain the severity of HIV infection and benefits of knowing individual and family HIV status and finally exploring the barriers and costs. This gave the individuals an in-depth understanding and correct information and potentially influenced their acceptance of testing services. A nationally representative study showed that up to 43.3% of Kenyans believed they were at low risk of contracting HIV [5] and saw no need of presenting themselves for HTC. Education and communication to all residents in simple terms and language in the individuals' household, a non- threatening environment may have increased perception of vulnerability without individuals feeling stigmatized and helped in promoting individual and collective responsibility witnessed by this high uptake of testing services. Household education included the effects of untreated HIV infection (threats) and the benefits of knowing ones HIV status as a gateway to care and treatment. During the educational sessions about HIV, it is important to explore and dispel myths and fears associated with HIV which are barriers and offer information on HIV prevention, care and treatment interventions available to those who test HIV positive which are benefits. The use of these four constructs of HBM in the household education, demonstrates that psychosocial theories can be affectively applied in HBTC and may have the potential of increasing demand for and uptake of services. Among the population that consented to testing, 39% tested for the first time demonstrating that there were individuals who experience barriers [25, 38] in accessing HTC services on their own. There were more men testing for the first time than females, this confirms that HBTC is an effective strategy in reaching men compared to other approaches [13, 25, 39].

Although the majority of the individuals had tested for HIV before, the HIV prevalence was significantly higher among those who had ever had an HIV test than those who were testing for the first time. This finding demonstrates that HBTC is effective in diagnosing HIV among persons who believe they are HIV negative on account of a previous negative test and who would not seek testing again and only benefit when services are taken to them. The finding may suggest that individuals who test HIV negative become complacent and do not take preventive measures against HIV acquisition or alternatively may have tested in the window period. One of the anticipated benefits of HBTC is to reach and test couples and families together to increase behavior change and promote access to care and treatment [40]. Although the finding of this study demonstrates a higher couple testing coverage than other approaches [13] it remains a challenge as only 19.2% coverage was achieved despite the efforts made by service providers to diversify service provision times. Most spouses were reported to be out of their homes during the day seeking jobs in the city. Flexibility is necessary and this program adopted to test partners of those already tested at a later date when they were available and encouraged partner mutual disclosure of HIV status [41]. Service provision challenges were mitigated by having a rigorous support supervision process and adhering to the national quality assurance measures and requirements [42]. A major limitation of this study was that there was no control group and therefore we cannot report cause and effect but limit our report to associations. Even though there was overwhelming support from the community shown by the increased uptake of services, we may not categorically conclude that the use of community participation principles and constructs of health belief model contributed to the outcome. Future work should examine the association between the use of constructs of the HBM and change in sexual behavior to avoid HIV risk.

## Conclusion

HBTC is an effective strategy to reach and test persons with low risk perception who may not access HTC services outside their homes as well as identify substantial number of persons who believe they are HIV free on account of previous negative result and link them early to care and treatment. HIV prevalence was higher among those who had ever tested (6.4%) than those who tested for the first time (4.0%). Through the use of a well-planned systematic HBTC approach based on community participation principles and the health belief model, it is possible to achieve measurable and high household coverage and increased uptake of HTC services in hard-to-reach urban populations.

### What is known about this topic

- HBTC is an acceptable strategy in reaching those who experience barriers in accessing HIV testing and counseling services;
- The strategy helps in diagnosing HIV infection early before the individual who is infected gets sick;
- Psychosocial models have been effectively used in other public health interventions to increase community participation, involvement and adoption of disease preventive strategies.

### What this study adds

- The use of community participation principles and health belief model constructs increases community acceptance and uptake of services and ensures community wide saturation with services, reaching those who have low HIV risk perception;
- Conducting a census in the target population and mapping of households allows for determination of a community wide denominator which helps in determining coverage and uptake of services;
- Engaging community members in gainful short term hires during the implementation is a motivator for mobilizing the community.

## Competing interests

The authors declare no competing interest.
